# Optimization of shunting operation plan in large freight train depot based on DQN algorithm

**DOI:** 10.1371/journal.pone.0320762

**Published:** 2025-04-08

**Authors:** Jiandong Qiu, Shusheng Xu, Minan Tang, Jiaxuan Liu, Hailong Song

**Affiliations:** 1 School of Mechanical Engineering, Lanzhou Jiaotong University, Lanzhou, China; 2 School of Automation and Electrical Engineering, Lanzhou Jiaotong University, Lanzhou, China; University of Tabuk, SAUDI ARABIA

## Abstract

Shunting operation plan is the main daily work of the freight train depot, the optimization of shunting operation plan is of great significance to improve the efficiency of railway operation and production and transportation. In this paper, the deep reinforcement learning (DRL) environment and model of shunting operation problem are constructed by three elements: action, state and reward, taking shunting locomotive as the agent, the lane number of the fall-down train group as the action, the fall-down conditions of the train group as the state, and design the reward function based on the total number of shunting hooks generated after the group’s descent and reorganization. The model is solved using the Deep Q network (DQN) algorithm with the objective of minimizing the number of shunting hooks, the optimal shunting operation plan can be solved after sufficient training. DQN is verified to be effective through example simulations: Compared to the overall planning and coordinating (OPC) method, DQN produces a shunting operation plan that occupies fewer lanes and produces 10% fewer total shunting hooks. Compared to the binary search tree (BST) algorithm, DQN produces 5% fewer total shunting hooks. Compared with the branch and bound (B&B) algorithm, DQN takes less time to solve, and the number of freight train removed by the coupling and slipping operations is reduced by 5.3% and 2.9%, respectively, and the quality of the shunting operation plan is better. Therefore, this paper provides a new solution for the intelligentization of shunting operations in large freight train depot.

## Introduction

The large train depot has a huge amount of annual train overhaul, more lines in the section, a large amount of freight trains in stock, and with the shunting operation, the storage location of the trains has been in a state of dynamic change [1]. If in accordance with the daily overhaul operation requirements, in the many shares of the many vehicles, select the train to be repaired, a larger number of shunting operations are required. And now the preparation of shunting operation plan, basically using manual way, according to the experience of the preparer to complete, whether there is optimization space, there is no reliable judgment standard. The large amount of shunting operations, brought about by low productivity, igh operating costs, increased safety risks and other issues [2], appears to be very prominent. The shunting operation has also become a safety critical point in the train depot.

Many scholars have conducted in-depth research on the problem of shunting operation planning. Song et al [3,4] put forward the concepts of locating the train group, must adjustable train group and passing adjustable train group, and from determining the position of locating the train group at each station in the train to be organized, he discusses the method of determining the whereabouts of the train group and the adjustable train group in the computerized preparation of train grouping hook plan. When the number of fall-down trains is not an integer power of 2, Song [5,6] used the analytical calculation method to propose the quantitative criteria for evaluating the shunting hook plan of the train grouping of the pick-and-hook train, i.e., converting the number of slipping hooks, effectively using the redundant counterparts and optimizing the screening of shunting plans. Gao et al [7] proposed the "Elimination of inverse-order", which realizes the optimization of shunting operation through the two phases of "inverse substitution" and "positive sequence establishment". This method is especially effective when the number of lines is limited. According to the principle of shunting operation, Wang et al [8] abstracted the train fall-down problem into a sequencing problem and proposed a binary tree-based planning method, which utilizes BST to obtain an ordered sequence of trains as a selection set for the fall-down scheme, and then screened according to the screening conditions to obtain the optimal fall-down scheme. Shi et al [9] proposed a second-level model of Lexicographic Goal Programming by considering flexible storage and column occupancy, which solves the problem of improving the quality of shunting operation planning for moving train stations under limited facility conditions. With the objective of reducing the number of lane occupancy during shunting operations and taking multiple constraints into consideration, Hu et al [10] designed a simulated annealing algorithm based on the generation of feasible paths for shunting operations and the exchange of operation priorities to solve the problem, which can obtain an optimized shunting operation plan in a relatively short period of time. Chen et al [11] pointed out the strong coupling of the collaborative preparation process of the technical operation plan of the high-speed railroad hub station and the shunting operation plan of the moving train station, and designed a hybrid optimization algorithm combining the bottleneck process, the heuristic allocation rule, and the parallel tabu search algorithm to solve the problem. Zhang et al [12] addressed how to efficiently prepare the shunting operation plan for picking trains by station, using a 0-1 linear programming model and a heuristic branch delimitation algorithm based on the elimination of the inverse rule to solve the problem of preparing the shunting operation plan for picking trains.

With the wave of technological innovation, the intelligent demand of railway shunting work is growing, and the traditional optimization algorithm has gradually appeared to be incompetent and difficult to meet the growing intelligent demand [13]. The emergence of reinforcement learning (RL), an emerging technology, brings new solutions and ideas for railway shunting work. It continuously optimizes the decision-making process through the interactive learning between the agent and the environment, and promotes the development of railway shunting work in the direction of more efficient and more intelligent.

RL is an important branch of machine learning [14,15], which is based on the idea that an agent learns optimal behavioral strategies through interaction with its environment. DRL is the product of combining deep learning (DL) with RL, which enables an agent to learn complex strategies through interaction with the environment in order to solve problems in high-dimensional and continuous action spaces. The rise of DRL can be traced back to the proposal of DQN, a milestone in the field of DRL as this work demonstrated for the first time that DL can be successfully applied to RL tasks. The DQN algorithm solves the scaling problem of the traditional Q-learning algorithms [16,17] in high-dimensional state spaces by estimating the Q-value using a deep neural network. The DQN algorithm was first proposed by the DeepMind team in 2013 [18] and improved in the 2015 paper [19] by attempting to reduce the dependency between the computation of the target Q-value and the parameters of the Q-network to be updated with two Q-networks, which demonstrates the application of the DQN algorithm to the Atari 2600 game, proving its surpassing of the human player’s capabilities.

In railway shunting, Hirashima [20,21] proposed a Q-learning based RL approach aimed at solving the problem of shifting and dispatching freights in a train through autonomous learning. Šemrov et al [22] proposed a Q-learning based train re-scheduling method, which is capable of efficiently adjusting the train’s operation plan to reduce delays and improve the reliability of the railway system in the event of major disruptions in train operations. References [23,24] have mainly used DRL methods to solve the train unit shunting problem faced by Dutch Railways. Shi et al [25] proposed an optimization method combining the table shunting method, RL and Q-learning algorithm, which is able to solve the quality approximation of shunting operation plan in a shorter time compared to the traditional method.

**Fig 1:**
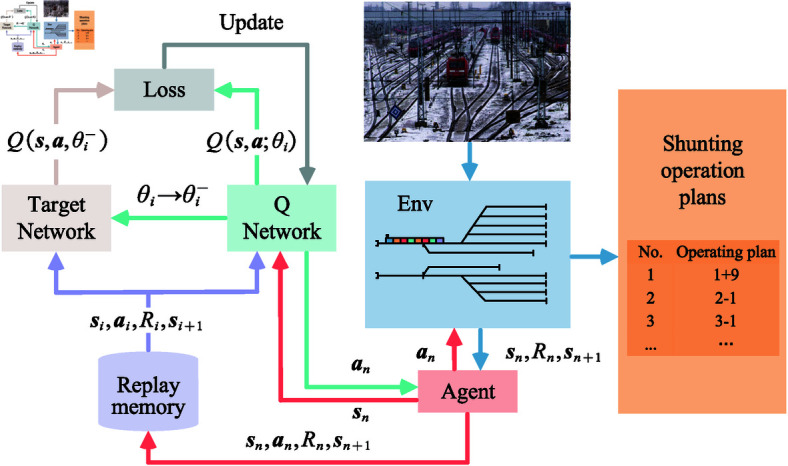
The process of generating a shunting operation plan. Considering the shunting yard as an environment for the reinforcement learning model to interact with, the agent learns continuously with the environment and finally outputs the shunting operation plan.

In summary, the development of artificial intelligence technology, especially the application of RL in optimization problems, provides a new way to improve the intelligence level of shunting operation plan. DQN algorithm, as a leader in RL, has already demonstrated its powerful optimization ability in many fields. However, when directly applied to the optimization of shunting operation plans for large freight trains, the DQN algorithm faces challenges such as huge state space, low learning efficiency, and weak strategy generalization ability. To address these issues, this paper proposes a DQN algorithm for the shunting operation characteristics of large freight trains, aiming to improve the optimization quality of the shunting operation plan by enhancing the exploration capability and learning efficiency of the algorithm. By introducing advanced experience playback mechanism, goal network and prioritized experience playback, the improved algorithm in this paper is able to learn more stable and effective shunting strategies in complex environments. Fig 1 shows a schematic diagram of the shunting operation plan generation process.

In this paper, DRL technology is applied to the preparation process of shunting operation plan for freight trains. Shunting locomotive is selected as agent, and a RL model is constructed through the three elements of actions, states and rewards, and neural networks are added on the basis of traditional Q-learning algorithm, which are used to replace the Q-table in Q-learning algorithm. By introducing the two key techniques of experience replay and fixed Q-targets, the deep neural network is used to approximate the state-action value function (Q-value), and the optimal shunting operation plan can be obtained when the Q-value converges. The algorithm can generate the shunting plan in an optimal way according to the current status of the freight stock in the train depot and the maintenance plan of the day, and reduce the number of shunting operations, thus reducing the cost and improving the efficiency and ensuring the safety. The main contributions of this paper include:

(1) Due to the large number of trains stored in large train depot and the large number of lanes, this paper proposes a kind of DQN algorithm suitable for the characteristics of shunting operations of large freight trains, by reasonably setting the action and state space of the environment and designing a set of reward mechanisms suitable for shunting operation optimization, in order to guide the algorithm to learn a more economical and efficient shunting plan.
(2) Verified by simulation experiments, the algorithm in this paper improves the performance in shunting operation plan optimization compared with OPC, BST and B&B, which provides a new solution and theoretical support for the intelligent scheduling of railway freight train depots.

The structure of this paper is as follows: Section 2 describes the process of preparing the shunting operation plan. Section 3 establishes a DRL model in conjunction with the problem. Section 4 solves the problem using DQN algorithms. Section 5 analyzes several cases with arithmetic examples. The last section summarizes the paper.

## Description of the problem

For the trains to be overhauled that are staying on the storage line of the train depot, the disordered and random groups of trains are organized into groups of a specific order in accordance with the grouping order of the overhaul plan. The process of group fall-down can be carried out using the shunting table, as shown in Table 1. The rows in the shunting table represent the lanes that can be used to park the units, and the columns in the table represent the mutual positions of the units in the train to be organized. It is assumed that the lead track is located on the left side of the shunting yard, and the left side of the freight train is the front and the right side is the rear. The shunting locomotive operates at the left end of the freight train, and connects the freight trains according to the order of the station. The order of the wagons is increasing from left to right.

**Table 1:** Fall-down plan table.

Lane number	Train number												
	4	6	1	4	6	1	2	5	3	2	3	1	4
*L*_1_			1			1						1	
*L*_2_							2			2	3		
*L*_3_									3				4
*L*_4_	4			4				5					
*L*_5_		6			6								

Connection: Starting from the front of the train, the number of neighboring groups is incremented or equal and the difference is not greater than 1.

Non-connection: the neighboring trains do not meet the conditions of connection of the group connection form.

Temporary joint sequence: the existence of a non-connection form of the group of trains in the sequence.

For example: a train to be organized in the train group numbered ’1,2,3,5,4,6,7’. The group of trains numbered 1 is adjacent to the group of trains numbered 2, the number is arranged in increasing order, and the difference in the number is not greater than 1, so the two groups constitute a connection form; The group of trains numbered 3 is adjacent to the group of trains numbered 5, which is arranged in increasing order, but the difference in numbering is 2 (greater than 1), so the two groups constitute a non-connection form; The group of trains numbered 5 is adjacent to the group of trains numbered 4, but the numbering is not in increasing order, so the two groups also constitute a non-connection form. The rest of the neighboring groups are connected. There are two non-connections in this train so the train is a temporary joint sequence.

**Fig 2:**
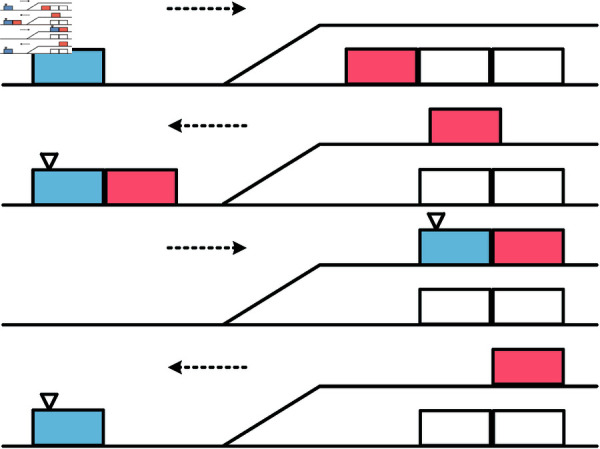
Schematic diagram of lead track shunting. The shunting locomotive is shown in blue and the group of cars to be organized is shown in red. The figure shows the process of completing a pull-out line shunting.

Adjustment of group order is realized through the shunting locomotive. The process of shunting locomotive to complete a process of attaching a group of trains or detaching a group of trains is called a shunting hook, which is divided into attaching hook and detaching hook. The number of shunting hooks directly affects the efficiency of shunting operations, so the essence of shunting operation plan optimization is to reduce the time of shunting operations to improve the efficiency of the operation by reasonably arranging the shunting hooks to achieve the minimum value. Fig 2 shows the schematic diagram of attaching and detaching hook in a lead track shunting.

## Deep reinforcement learning modeling

The shunting operation plan is divided into 2 parts: fall-down and reorganization, and the RL model is constructed by setting up three elements: action, state and reward.

### Parameter definitions

Some of the variables in the model that will be developed in this paper are now explained as shown in Table 2.

**Table 2:** Variables used in this paper.

Variables	Definition
*L*	Number of lanes available for shunting at the yard.
$L_{i}$	Lane *i* for shunting.
*G*	Attaching hook.
*Z*	Detaching hook.
*D*	Shunting hook.
*N*	Number of trains in the initial train to be organized.
*E*	Number of the rightmost train group connected to the shunting locomotive.
$P_{ij}$	Train *j* on the lane *i* in the shunting table.
$C_{i}$	Number of the leftmost train group on the lane *i*. If there is no train group in that lane, the nonexistent.

### Reorganization process after the fall-down

After the completion of the fall-down, the trains on the shunting yard are reorganized to realize the requirements of grouping according to the station. The group reorganization is realized by attaching and detaching the trains after the fall-down. The conditions of attaching, detaching and the process of group reorganization are as follows [25].

(1) Attaching conditions

Condition 1: Both $P_{ij}$ and its left end group can form a connecting form with the group attached to the shunting locomotive and there is no group with a larger number than $P_{ij}$ in the lane.

Condition 2: Search for the temporary joint sequence in descending order of the number of disjunctions, where there exists $P_{ij}=C_{m}$ or $P_{ij}=C_{m}+1$ , and *i* ≠ *m*.

Condition 3: $P_{ij}=C_{m}$ or $P_{ij}=C_{m}+1$ , and *i* ≠ *m*, exists in all connecting form groups.

When any of the above conditions for attaching trains are met, attach $P_{ij}$ and its left train group to the shunting locomotive.

(2) Detaching conditions

Condition 4: There exists $C_{i}$ such that $E=C_{i}$ .

Condition 5: There exists $C_{i}$ such that $E=C_{i}+1$ .

When any of the above conditions for attaching trains are met, the rightmost train group connected to the shunting locomotive will be slipped to the corresponding lane.

(3) Train group reorganization process

**Fig 3:**
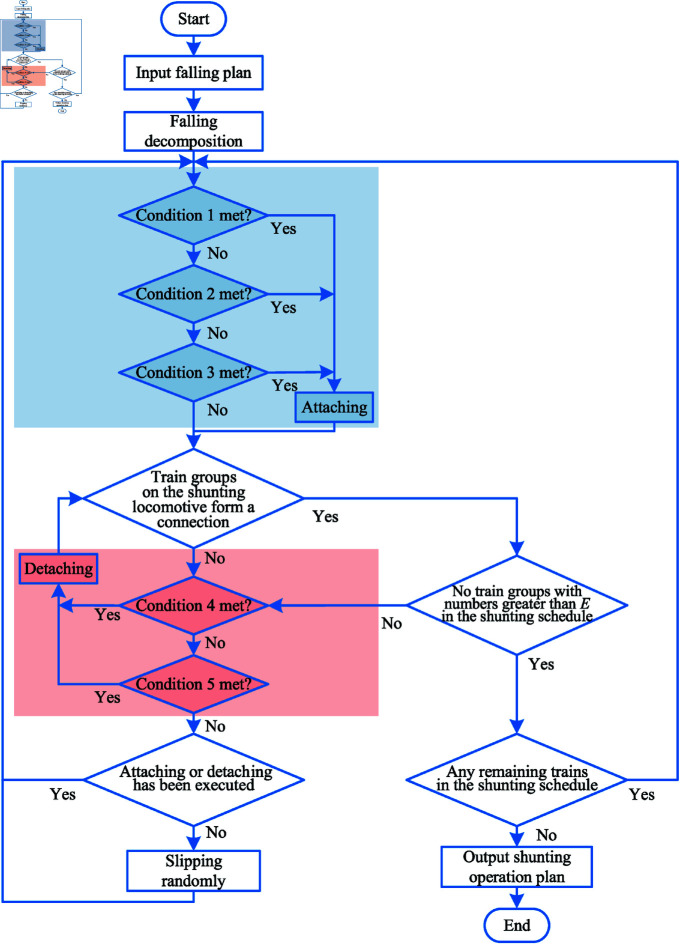
Reorganization process after train group falling. The blue color is for attaching conditions and the red color is for detaching conditions.

The reorganization process of the car group is shown in Fig 3. After the completion of the fall of the train, the rest of the train group need to first according to the shunting table in order to determine the attaching conditions 1-3, to determine whether attach trains. Then determine whether the train group on the shunting locomotive constitutes a connection, whether there is no train group in the shunting table with a larger number than *E*, and there are remaining train groups in the shunting table will continue to determine the attaching conditions until they are not met. Then, according to the conditions 4-5 to determine whether to detach the train, slip the train group until all the train groups on the shunting locomotive form a connected form, and there is no train group with a larger number than *E* in lane. At this time, if this round has not been attached or detached operations, the *E* group of trains randomly slipped to any lane, and once again to determine the attaching and detaching conditions. Cycle the above process until there is no train group in the shunting table, and finally get the shunting operation schedule, and get the number of attaching hooks and detaching hooks according to the schedule.

### Action and state of RL

Agent is subject in RL, this paper chooses to take the shunting locomotive as the agent, take the shunting yard as the environment, take the fall-down situation of all the train groups in the shunting yard as the current state, take the number of the train in the current state in the lane to be falling to as the action of the agent, the agent executes the action to interact with the environment, so as to change the state of the environment.

Use a *N*-dimensional row vector $a_{n}$ to represent the agent’s action on the nth train group (1 ≤ *n* ≤ *N*), with the *n*th component recorded as $a_{n}$, which indicates the lane number selected by the agent. The rest of the train groups do not execute the falling action, so the rest of the components are 0. For example, if the 2nd car group falls to lane number 1, then $a_{n}=\left(0,1,0,\cdots \;,0\right)$. If there are *L* (the number of lanes available for shunting) actions that can be selected by the agent, then the set of actions that can be executed by a train group is A= {1,2,⋯,L}.

Because the shunting yard is used as the environment, and the shunting yard can be abstracted into a shunting table to represent the falling situation of the train group. The shunting table is simplified as an *N*-dimensional row vector $s_{n}=\left(x_{1},x_{2},\cdots \;,x_{N}\right)$, which is used to represent the state of the shunting table after the nth train group fell down. Here, $x_{n}$ represents the lane number where the *n*th train group is located after it has fallen. When all train groups have fallen, the initial state of the shunting table is $s_{0}=\left(0,0,\cdots \;,0\right)$. When the *n*th train group in the train column has completed its fall, the value of $x_{n}$ changes. For example, after the second train set falls onto lane number 1, then $s_{n}=\left(2,1,0,\cdots \;,0\right)$. After the agent executes the action $a_{n}$, the state is updated, the formula is as follows:


$$\begin{array}{ccc} s_{n+1}=s_{n}+a_{n} & & \end{array}$$ (1)

Where, $s_{n+1}$ represents the updated state. $s_{n}$ represents the state before the update. $a_{n}$ represents the action taken for the *n*th train group.

### Reward function design

The merit of the shunting operation plan is mainly measured by the number of shunting hooks. The Q-value obtained from the accumulation of rewards after the execution of actions by the agent converges stably to a maximum value, indicating that the current algorithm has found the optimal shunting plan. The reward function is designed based on the connection status after the current train group has fallen and the total number of shunting hooks generated after the completion of the fall. The reward is divided into two parts: immediate reward and delayed reward.

Immediate rewards are determined based on the state of the composition of the current train group with the train group on the lane after the train group has completed its fall-down. The immediate reward $r\left(s_{n},a_{n}\right)$ is as follows:


$$\begin{array}{ccc} r\left(s_{n},a_{n}\right)=\sigma & & \end{array}$$ (2)

Where, *σ* is a 0-1 variable, which takes the value of 1 when the current train group completes its fall and forms a connected state with the train group on the lane, and takes the value of 0 otherwise.

After the train group completes its fall and reorganization, the delay reward is determined based on the total number of shunting hooks generated [26,27]. The delay reward $r_{d}$ is as follows:


$$\begin{array}{ccc} r_{d}=\frac{\lambda }{D} & & \end{array}$$ (3)

Where, *λ* is any positive number.

The immediate and delayed rewards are combined to form the cumulative reward obtained by the agent after executing the action $a_{n}$. The cumulative reward $R\left(s_{n},a_{n}\right)$ is as follows:


$$\begin{array}{ccc} R(s_{n},a_{n})=\{\begin{array}{cc} \overset{n}{\underset{i=0}{\sum }}r(s_{i},a_{i})\; & n\neq N\\ {}[2ex]\overset{n}{\underset{i=0}{\sum }}r(s_{i},a_{i})+r_{d}\; & n=N \end{array} & & \end{array}$$ (4)

Where, $R(s_{n},a_{n})$ is the cumulative reward received by the agent for executing the action $a_{n}$ while in state $s_{n}$. *i* is a natural number from 0 to *n*.

## Problem solving

The expectation of the reward that the agent can get after executing the action is represented by the Q-value, and the larger the Q-value is, the better the current strategy is. When solving the model, the mapping relationship between the train and the optimal shunting operation plan is constructed with the goal of minimizing the shunting hooks. The completion of the falling of all train groups to be assembled is recorded as one episode. In each episode, at state $s_{n}$, the *n*th train group executes action $a_{n}$ to complete the falling, and after the action is executed, an cumulative reward $R(s_{n},a_{n})$ is obtained. The Q-value is updated based on the cumulative reward. The formula for updating the Q-value is as follows:


Qn′ (sn,an)←Qn (sn,an)+β [R (sn,an)+γmax ⁡ an+1∈AQn+1 (sn+1,an+1)−Qn (sn,an)] (5)

π(a|s)= {1−εa= argmax ⁡ Q(s,a)εa≠ argmax ⁡ Q(s,a),ε=1F (6)

Where, $Q_{n}^{\prime }\left(s_{n},a_{n}\right)$ is the updated Q-value of the agent after executing the action $a_{n}$ in state $s_{n}$, where $a_{n}$ is determined under the guidance of policy *π* ( *a* | *s* ) , which represents the probability of the agent choosing action *a* in state *s*. *β* is the learning rate, β∈ (0,1]. The higher the learning rate, the greater the proportion of results obtained from new trials, and vice versa. *γ* is the discount factor, γ∈ (0,1]. If is closer to 0, the agent will be more inclined to immediate rewards. If *γ* is closer to 1, the agent will consider future rewards more. *ε* is the exploration rate of the agent. *F* is the number of episodes. *argmax* ⁡  *Q* ( *s* , *a* )  is the action that maximizes the Q-value in state *s*. At the beginning, when the number of episodes is low, *ε* is higher, which allows for significant progress and learning. As the agent learns about future rewards, *ε* decays, which facilitates the discovery of higher Q-values.

**Fig 4:**
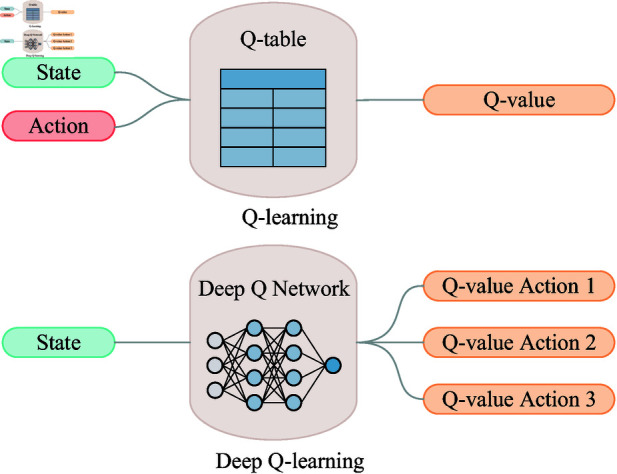
Comparison of Q-learning and DQN. A schematic of the Q-learning algorithm is shown at the top, and a schematic of the DQN algorithm is shown at the bottom.

The traditional Q-learning algorithm stores the Q-values in a Q-table and updates them according to formulas (5)-(6), which is the learning process of the agent. The DQN algorithm replaces this Q-table with a neural network, as shown in Fig 4 [28]. However, directly and simply combining the two can lead to two obvious problems:

(1) Neural networks require that the input samples be independent of each other, unrelated, and satisfy the independent and identically distributed (i.i.d.) condition. However, the states input in RL are interrelated and do not meet the i.i.d. condition.

(2) The introduction of nonlinear functions, using neural networks to approximate the Q-table, may lead to non-convergence of the training results. For example, in regression problems, if there is correlation between the input data, it may cause the function fitted by the network to change, resulting in inaccurate predictions and a high loss.

The following two major improvements alleviate the problem of network convergence difficulties when input data do not meet the independent and identically distributed (i.i.d.) condition.

(1) Experience Replay

The memory in experience replay is used to store past experiences. Since Q-learning is an off-policy, oﬄine learning method, it can learn from current experiences, past experiences, and even the experiences of others. Therefore, incorporating previous experiences randomly during the learning process can make the neural network more efficient.

Thus, experience replay solves the problem of correlation and non-stationary distribution. It stores the transition samples $s_{n},a_{n},R_{n},s_{n+1}$ obtained from the agent’s interaction with the environment into the replay memory network [29]. When training, it randomly takes out a batch for training, thereby disrupting the correlation within. By using experience replay, the advantages of off-policy can be fully utilized, where the behavior policy is used to collect experience data, and the target policy focuses solely on value maximization.

(2) Fixed Q-targets

The Q-target serves as a mechanism to break the correlation. Using Q-targets results in two networks within the DQN algorithm that have the same structure but different parameters. The Q network uses the most up-to-date parameters, while the target network uses the previous ones. $Q\left(s,a,\theta_{i}\right)$ represents the output of the current Q network, which is used to evaluate the value function of the current state-action pair. $Q\left(s,a,\theta_{i}^{-}\right)$ represents the output of the target network, which can be used to calculate the target Q and update the parameters of the Q network based on the loss function. After a certain number of episodes, the parameters of the Q network are copied to the target network. For example, you can train the Q network for 10 episodes, then assign the updated parameters to the target network, and then train the Q network for another 10 episodes, and continue this process repeatedly. After introducing the Target Network, the target Q-value remains constant for a period of time, thereby reducing the correlation between the current Q-value and the target Q-value, and enhancing the stability of the algorithm.

The Q-value update formula and policy are the same as the Q-learning update formulas (5)-(6) mentioned earlier, and the neural network parameters $\theta_{i}$ and $\theta_{i}^{-}$ are added to the aforementioned formulas [19], the formula is as follows [30,31]:


Target Q=R (sn,an)+γmax ⁡ an+1∈AQ (sn+1,an+1,θi−) (7)

$$\begin{array}{ccc} Q^{\prime }(s_{n},a_{n},\theta_{i})\leftarrow Q(s_{n},a_{n},\theta_{i})+\beta [\text{Target }Q-Q(s_{n},a_{n},\theta_{i})] & & \end{array}$$ (8)

π(a|s)= {1−εa= argmax ⁡ Q(s,a;θi)εa≠argmax ⁡ Q(s,a;θi),ε=1F (9)

The loss function of the DQN is expressed as follows [32]:


$$\begin{array}{ccc} L_{i}\left(\theta_{i}\right)=E_{s_{n},a_{n},R_{n},s_{n+1}}\left[{\left(\text{Target }Q-Q\left(s_{n},a_{n},\theta_{i}\right)\right)}^{2}\right] & & \end{array}$$ (10)

Stochastic gradient descent is used to update the neural network parameters $\theta_{i}$. The gradient of the loss function is as follows:


$$\begin{array}{ccc} {▿}_{\theta_{i}}L_{i}\left(\theta_{i}\right)=E_{s_{n},a_{n},R_{n},s_{n+1}}\left[\left(\text{Target }Q-Q\left(s_{n},a_{n},\theta_{i}\right)\right){▿}_{\theta_{i}}Q\left(s_{n},a_{n},\theta_{i}\right)\right] & & \end{array}$$ (11)

**Fig 5:**
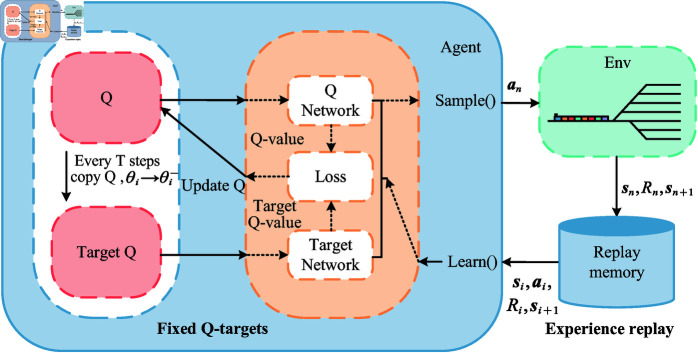
Flowchart of DQN algorithm. The agent generate data by interacting with the environment and the obtained data is stored in the replay memory and the data is used for the training of the neural network.

As shown in Fig 5, the agent first continuously interacts with the environment to obtain interaction data $s_{n},a_{n},R_{n},s_{n+1}$, which is stored in the replay memory. When there is enough data in the experience pool, a batch size of data is randomly taken out. The Q prediction value is calculated using the current network, and the Q target value is calculated using the target network. Then, the loss function between the two is calculated, and the gradient descent is used to update the current network parameters. After repeating this several times, the parameters of the current network are copied to the target network.

Processing techniques such as experience replay and fixed Q-targets have been added to the DQN algorithm [33]. The DQN algorithm design steps are as follows:

Step 1: Initialize the memory *D*, initialize the Q_eval_net (current data), and initialize the Q_target_net (historical data).

Step 2: Preprocess the environment and input the state *s* into the DQN.

Step 3: Select an action using an *ϵ*-greedy  (0<ε<1) policy: with probability *ϵ*, we choose a random action a (a∈A), and with probability 1 − *ε*, we select the current optimal action based on the model, that is, a= argmax ⁡ Q(s,a;θi) (where $\theta_{i}$ represents the parameters of the neural network).

Step 4: The train group executes the selected action *a* in state *s*, and transitions to a new state $s_{n+1}$ with the reward $R\left(s_{n},a_{n}\right)$.

Step 5: Store the state information in the memory *D*, denoted as $s_{n},a_{n},R_{n},s_{n+1}$.

Step 6: Randomly select a batch of samples from *D*, denoted as $\left\{s_{i},a_{i},R_{i},s_{i+1}\right\}$. In Q_target_net, calculate the true Q-value (Q_target ($s_{i+1}$)) for the next state $s_{i+1}$ using the Q-learning algorithm. In Q_eval_net, calculate the Q-value (Q_eval ($s_{i}$))for the current state. Use mean squared error to calculate the loss and execute gradient descent to minimize the loss.

Step 7: Replace the parameters of Q_target_net at regular intervals.

Step 8: Repeat steps 2 to 7 for *M* rounds.

## Case analysis

To verify the effectiveness of the algorithm, the following case study analysis was conducted in this paper. The experimental operating environment is as follows: Equipment: CPU: Intel Core i5 2.50 GHz, IDE: PyCharm, Development language and tools: Python 3.11.9 and Pytorch 2.3.1. Based on the current storage state of freight trains in the depot and the daily maintenance plan, the most optimized shunting plan is generated.

The following is an example of the layout of the lines in a train depot in Lanzhou to illustrate the functional division of a freight train depot. Generally speaking, most of the train depots are only responsible for depot repair work, and only a few train depots will share part of the station repair task if their own equipment is sufficient and the space of the site permits.

**Fig 6:**
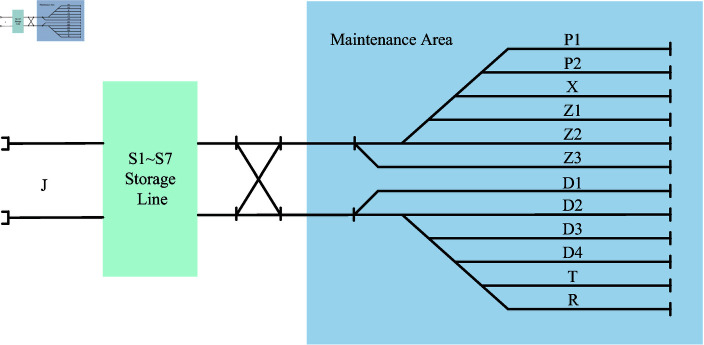
Schematic layout of a train depot in Lanzhou. The left side is the storage line, and the right side is the maintenance area.

The layout of the train depot can be referred to Fig 6, in which a number of lanes areas with different functions are planned inside the depot. Among them, D1-D4 belongs to depot repair operation area, which mainly accomplishes depot repair operation of trains and ensures that the key components and overall performance of trains are repaired and improved. Z1-Z3 is the station repair operation area, which mainly accomplishes the station repair task of trains, and deals with some relatively minor problems that need to be repaired quickly in the process of train operation. T is the girder adjustment area, whose core function focuses on the precise adjustment of the girders of trains to ensure the stability of the train structure. X is the tanker cleaning operation area, which removes toxic, flammable and explosive gases or liquids that may remain, so as to build a solid foundation for the subsequent safety inspection and repair. P area is the operation area of shot blasting and painting, which is responsible for shot blasting and polishing as well as painting to beautify and protect the train compartments. Area R is the pre-maintenance operation area, which carries out preliminary inspection and pre-maintenance for every train coming into the train depot detecting hidden dangers and formulating maintenance strategies in advance. S1-S7 is the storage area for trains, which mainly carries out the operations of transferring, staying and preparing trains in the depot. J area is used as the access line of the train depot to ensure the smooth entry and exit of trains.

### Computational process

**Fig 7:**
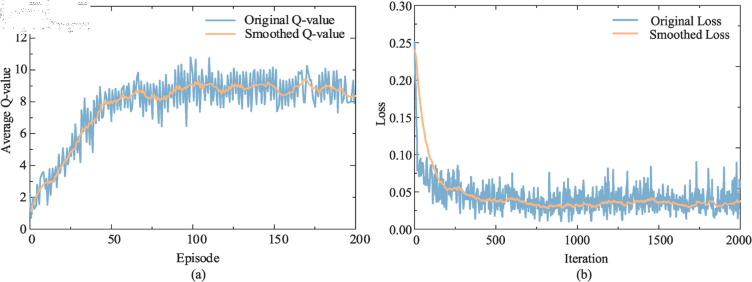
Training curve chart. (a) Q-value change curve during the training process. (b) Loss value change curve during the training process.

Taking the previously mentioned train column to be assembled ’4,6,1,4,6,1,2,5,3,2,3,1,4’ as an example, when there are 5 available lanes (*L* = 5), after the RL model has been fully trained, the changes in Q-value and loss value are shown in Fig 7.

Initially, the Q-values are zero, and the agent’s exploration rate is high to learn various plans. As the number of training episodes increases, the Q-values continuously increase. After the agent has fully learned, the Q-values tend to stabilize, reaching a state of convergence. At this point, the sequence of actions executed by the agent, when the Q-value is maximized, represents the optimal solution. After sufficient learning, the optimal shunting table obtained by the agent is shown in Table 3.

**Table 3:** Fall-down plan table after training.

Lane number	Train number												
	4	6	1	4	6	1	2	5	3	2	3	1	4
$L_{1}$	4			4				5					
$L_{2}$		6			6								
$L_{3}$			1			1	2			2			
$L_{4}$									3		3		4
$L_{5}$												1	

**Table 4:** Comparison of shunting operation plans obtained by different algorithms 1.

OPC		BST		DQN	
*L* = 4		*L* = 4		*L* = 3	
Serial number	Operating plan	Serial number	Operating plan	Serial number	Operating plan
1	4+14	1	4+16	1	1+14
2	3-1	2	2-1	2	2-1
3	1-1	3	4-2	3	3-2
4	4-1	4	3-1	4	2-3
5	3-2	5	2-1	5	3-1
6	2-1	6	4-2	6	2-2
7	4-1	7	1-1	7	1-2
8	2-2	8	2-1	8	2-2
9	4-2	9	1-2	9	1-1
10	1-1	10	4-2	10	2+5
11	2-1	11	3-1	11	1+6
12	4+7	12	1-1	12	2-1
13	3-1	13	4+5	13	3-1
14	1-1	14	2-3	14	2+4
15	3-1	15	4-3	15	3-11
16	1-2	16	1+4	16	1-1
17	3-3	17	4+5	17	3+15
18	2+4	18	2+6	18	1+1
19	3+8	19	3+2	19	
20	1+5	20		20	
*G*	5	*G*	6	*G*	5
*Z*	15	*Z*	13	*Z*	13
*D*	20	*D*	19	*D*	18

### Effectiveness comparison

Through the following two cases, the shunting plans generated by the algorithm in this paper are analyzed and compared with those produced by OPC, BST, and B&B, thereby verifying the effectiveness and superiority of DQN.

#### Case 1.

Select the train ’4,2,5,3,1,2,4,4,4,6,2,7,7,5,6,1,8,6’ [8], the use of DQN, BST and DQN shunting operation plan for comparison and verification, the results are shown in Table 4. Compared with OPC, DQN in the occupied lanes under the premise of equality, the total number of hooks generated by the shunting operation of DQN is reduced by 2 hooks. Compared with BST, DQN in the occupied lanes under the premise of equality, the total number of hooks generated by the shunting operation of DQN is reduced by 1 hook. It can be seen that, under the premise of other conditions are the same, this paper’s algorithm obtains the shunting operation plan with fewer hooks, so the shunting operation plan is better.

**Table 5:** Comparison of shunting operation plans obtained by different algorithms 2.

OPC				B&B				DQN			
*L* = 4				*L* = 3				*L* = 3			
Serial number	Operating plan	Number of trains moved by coupling operations	Number of trains moved by slipping operations	Serial number	Operating plan	Number of trains moved by coupling operations	Number of trains moved by slipping operations	Serial number	Operating plan	Number of trains moved by coupling operations	Number of trains moved by slipping operations
1	1+9	9		1	1+9	9		1	1+9	9	
2	2-1		17	2	2-1		17	2	2-1		17
3	3-1		15	3	3-5		11	3	3-3		13
4	2-1		13	4	2-1		5	4	2-2		8
5	4-1		11	5	1-1		3	5	3-1		5
6	3-1		9	6	3+4	6		6	1-1		3
7	2-1		7	7	2-1		9	7	3+3	5	
8	4-1		5	8	1-1		7	8	2-1		7
9	1-1		3	9	3-1		5	9	1-2		4
10	2+3	5		10	2+3	7		10	2+4	6	
11	3-1		7	11	3-1		9	11	3-2		8
12	4-3		3	12	1-4		4	12	1-3		3
13	3+3	3		13	3+3	3		13	3+3	3	
14	4+5	11		14	1+7	13		14	1+7	13	
15	1+2	18		15				15			
Total	15	46	90	Total	14	38	70	Total	14	36	68

**Table 6:** Comparison of results of different algorithms.

Algorithm	Case1		Case2			
	Shunting hooks	Lanes occupancy	Shunting hooks	Lanes occupancy	Number of trains moved by coupling operations	Number of trains moved by slipping operations
OPC	20	4	15	4	46	90
BST	19	4	-	-	-	-
B&B	-	-	14	3	38	70
DQN	18	3	14	3	36	68

#### Case 2.

Select the train ’1,5,4,3,2,5,3,2,1,3’ [12], the use of traditional shunting operation algorithm and the algorithm derived from this paper shunting operation plan for comparison and verification, the results are shown in Table 5. Compared with OPC, the operation plan generated by DQN generates fewer shunting hooks and occupies fewer lanes, and the number of trains removed from the coupled operation and the number of trains removed from the slipped operation are reduced by 21.7% and 32.4%, respectively. Compared with B&B, the total number of shunting hooks generated from the shunting operation plan is the same under the premise of occupying the same number of lanes, but the number of trains removed by the couple operation and the number of trains removed by the slip operation in the method of this paper are reduced by 5.3% and 2.9%, respectively. Moreover, under the premise that the experimental equipment of this paper is not as good as that of B&B (CPU: Intel Core i7 3.4 GHz), the solution time of DQN is far less than B&B, compared with B&B of 1076s.

**Fig 8:**
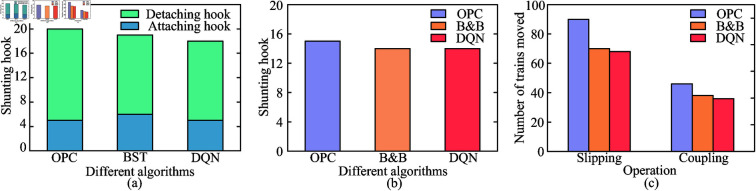
Comparison of different algorithms. (a) Different algorithms for shunting hooks in Case 1. (b) Different algorithms for shunting hooks in Case 2. (c) The number of trains removed by different algorithms in Case 2 for the coupling and slipping operations.

Table 6 and Fig 8 summarize the results of Case 1 and Case 2, which demonstrate the superiority of DQN by comparing the different algorithms.

## Discussion

### Innovations

In this study, the proposed algorithm is compared and analyzed with the OPC, BST and B&B in generating shunting operation plans through two specific examples, aiming to explore the performance of this algorithm and its significance and value in practical applications.

It is obvious from the results of the algorithm that the DQN shows significant advantages in the key indicators of shunting operations. First of all, in terms of the total number of hooks, both compared with OPC and BST, there is a reduction. This means that in the actual shunting operation, the use of DQN can reduce the operation steps, thus improving the operation efficiency and reducing the cost of labor and time. This advantage comes from the fact that DQN adopts a more optimized strategy when dealing with the sequence of train programming and lane utilization, which is able to plan the moving path of trains more accurately, avoiding unnecessary shunting operations and thus reducing the total number of hooks.

In Case 2, compared with OPC, this algorithm not only performs better in the number of shunting hooks, but also trains better results in the number of occupied lanes as well as the number of trains removed by the connecting and slipping operations. This further proves that DQN has stronger optimization ability when considering various shunting operation factors, and can ensure the smooth operation while reducing the occupation and waste of resources and improving the overall operational efficiency of railroad shunting yards.

The comparison with B&B is equally revealing. Although in some cases, such as the total number of shunting hooks, they are equal, the DQN still achieves a certain degree of reduction in the number of trains removed in both the coupling operation and the slipping operation. Moreover, it is worth noting that the solution time of the DQN is significantly reduced under the condition that the performance of the experimental equipment is not as good as that of the B&B. This indicates that DQN has an obvious advantage in computational efficiency and can provide a high-quality shunting operation planning scheme in a shorter time. This is of vital significance for the scenarios that require rapid response and frequent adjustment of operation plans in actual railroad transportation, which can effectively improve the timeliness and flexibility of railroad transportation.

### Suggestions

This study also has some limitations. Although the selection of cases is representative, it may not be able to cover all possible shunting operation scenarios and situations. Future research can further expand the scope and diversity of the examples, including different sizes of trains to be made up, complex railroad yard layouts, and various special operational requirements, in order to more comprehensively verify the stability and adaptability of the algorithm in this paper.

In addition, although DQN shows superiority in the current comparison, with the continuous development of railroad transportation technology and the emergence of new operational requirements, there is still a need to continue to pay attention to and research on other possible improvement directions and optimization strategies, in order to continuously improve the performance of the algorithm, so that it can better adapt to the future development trend of railroad transportation, and provide stronger support for the efficient and safe operation of railroad shunting operations.

In conclusion, through the in-depth analysis of the cases, DQN shows the effectiveness and superiority in shunting operation plan generation, but also points out the direction for future research, which needs to be improved and optimized in a wider range of scenarios and longer-term development.

## Conclusion

In this paper, a large freight train shunting operation plan optimization method is proposed on the basis of combining the table shunting method and DQN algorithm.

(1) A DQN algorithm is designed to address the characteristics of shunting operations of large freight trains, and a DRL model of the shunting operation problem of large freight trains is established by building an action and state space that conforms to the shunting environment and designing a suitable reward function, which transforms the shunting operation plan optimization problem into the problem of finding the optimal pair of dropping schemes. The mapping relationship between the train and the optimal shunting operation is established by taking the minimum number of shunting hooks as the objective, and the optimal shunting operation plan is obtained when the cumulative Q-value tends to be stable and reaches the convergence state.

(2) The reasonableness and effectiveness of this paper’s algorithm is verified through simulation experiments. Compared with OPC and the BST, DQN occupies fewer lanes and the shunting operation plan is better. Compared with the B&B, DQN can solve the shunting operation plan in less time, and under the premise that the number of shunting hooks is the same, the shunting operation plan obtained by this paper’s method is of better quality. In addition, the quality of the shunting plan obtained in this paper is better under the same number of hooks. The improved performance of this paper’s method in shunting operation plan optimization provides a new solution and theoretical support for the intelligence of shunting operation in large freight train depot.
